# Atlanta Academy of Medicine

**Published:** 1875-07

**Authors:** 


### Atlanta Academy of Medicine.

ROBERT BATTEY, M.D., Reporter.

Atlanta, May 18, 1875.

Dr. J. M. Johnson, President, in the chair.

Dr. Battey read a communication from a correspondent, detailing experience in spaying sows.

Dr. J. M. Johnson asked if care was used in ovariotomy to remove all blood from the cavity.

Dr. Battey replied that it was a rule with ovariotomists to use special care for the removal of blood as well as other fluids from the peritoneal sac. It is generally believed that effusions of blood into the peritoneum are not absorbed with the readiness here as in other cavities. He instanced a case of effusion of a quart, or even more, of blood by a stab, into the pleural sac, which was promptly and completely absorbed. Effusions of blood into Douglas' fossa (retro-uterine hæmatocele) are usually bridged over by a fibrinous exudation and not infrequently undergo absorption. He thinks that blood free in the abdominal cavity is very liable to undergo decomposition, break down and resolve itself into septic materials, which by absorption poison the system and produce septicæmia.

Dr. Johnson said that he had, thirty years ago, treated a man who had been stabbed in the iliac region. The man pursued his adversary a mile or more after receiving the injury. There was no external hemorrhage, but a considerable mass of omentum protruded at the wound, and so remained for four or five days. When seen there had been evidence of internal hemorrhage, as shown by tumefaction and tenderness. The protruding omentum had become discolored and lifeless, and was cut off and the stump returned. The patient made a speedy recovery, without an unpleasant symptom. He is aware of the care which is used to remove all blood from the abdomen in ovariotomy, but he is inclined to the opinion that the septic poisoning arises from constitutional defects rather than the mere effused blood which may be left behind. Adjourned.

---

J. B. BAIRD, M.D., Reporter.

---

Atlanta, May 25, 1875.

Dr. J. M. Johnson, President, presiding.

Dr. Battey presented a dysmenorrhœal membrane passed a few days ago by a lady aged thirty. It is an accurate cast of the cavity of the uterus, from the os to the fundus. The patient represents that she has passed similar membranes at every menstrual period for six years. Has been under his observation for nearly a year. The pain is relieved by dilating the cervix with sponge tents prior to the appearance of the menses. His object in bringing this specimen before the academy was to request that it be referred to Drs. Baird and Simpson for microscopical examination, and report.

He also exhibited the placenta and cord of a recently-delivered stillborn child. The placenta is healthy. The cord is deeply congested and livid, and presents, at a point about four inches distant from the umbilical extremity, a firmly-drawn knot, which has interrupted the fœtal circulation. Two weeks previously the movements of the child had been vigorous, and even boisterous. These movements were observed to diminish in frequency and force until there was a mere quiver occasionally felt, and ceased altogether ten days prior to labor. At birth the child was much blanched, macerated, and the cuticle easily detached. The mother thought herself within two weeks of term, but the child did not present a development of more than eight months. The knot in these cases doubtless occurs by the child passing through a loop accidentally formed by the cord.

Dr. W. S. Armstrong presented a tumor that he had removed about ten days before from the uterus of a lady. He desired, however, to state, in the outset, that it was due to the courtesy of Dr. H. V. M. Miller that he operated—the Doctor being present and participating in the operation. He said that he was sorry the gentleman was not present to participate in the report. But his indifference about reporting even his most interesting operations was well known. The tumor is fibroid in character, and a little less in size than his fist; it is shaped somewhat like an egg, and was attached to the fundus of the uterus by a pedicle one inch in diameter. The history of the case is as follows: She says her age is forty-two years. About two and a half years ago she commenced to have irregular and profuse menstruation, but thought it climacteric menorrhagia. These hemorrhages rendered her anæmic and kept her in a state of great prostration. So sure was she that it was her climacteric period, she never allowed an examination per vaginam to be made until it was done a few days ago by Dr. Miller. In the early part of the spring she was out at sea thirteen days, during most of which time it was storming. Violent sea-sickness came on, and the hemorrhage, from straining efforts at vomiting, was so great that she was not expected to survive. She says that she made a digital examination herself at this time, discovered the mouth of the uterus dilated and a tumor within—a condition which had not existed before. After her sea-voyage was over she reached Atlanta, and was examined by Dr. Miller and himself. They found the mouth of the womb of a size sufficient to admit two of the fingers, with the tumor presenting. The finger could easily be passed around the tumor, and up to its attachment at the fundus. At this and subsequent examinations the hemorrhage from the tumor was large. The mucous envelop was evidently the source from which it flowed. Patient was placed under the influence of ether, a pair of strong forceps guided by the finger introduced, and the extremity of the tumor seized. Traction was made and continued until more than half of the tumor was external to the uterus. The ecraseur was then applied around the tumor, and guided to the fundus of the uterus by the index finger. In seven minutes more the operation was completed. There was no loss of blood from the stump. He has visited her from time to time since the operation, and she has had no fever or experienced any tenderness in the uterine region.

Dr. Battey said the reference to the kind of anæsthetic used suggested the important subject of anæsthesia. He is inclined to the opinion that the surgeons of the South are behind the times on the subject of anæsthetics in their general and great preference for chloroform over ether. Is satisfied that chloroform is dangerous. Never lost a life from it, but has often seen alarming symptoms. Has never felt anxiety from ether, except in one case, and then his apprehension was not shared by his assistants. True, there are objections to ether, but they are overbalanced by the greater safety. Thinks these objections may be summed up under three heads: Firstly. Its difficult portability, being very volatile, especially in warm weather. It is bulky, a large amount being required to affect the patient. These objections pertain particularly to country practice. Secondly. The length of time required to anæsthetize the patient. This objection does not spring from any intrinsic demerit in the agent, but from want of skill in the administration. Dr. J. Marion Sims recently etherized a patient for him in less than five minutes. The ether acted as promptly as chloroform. It should be forced rapidly upon the patient from the first, and thus economize both time and ether. Thinks this method safer, as the blood is less saturated with the ether than when tardily administered.

Dr. Bozeman inquired if he had ever used a combination of ether and chloroform.

Dr. Battey replied that he thought the combination very objectionable, from the different volatility of the two substances. The chloroform gradually accumulates upon the sponge, and thus the more powerful and dangerous agent is freely administered in the later stages of the operation.

Dr. Baird said the length of time required to etherize a person depends largely on individual susceptibility and the quality of ether employed. Squibbs' ether acts much more promptly, and in far smaller quantity, than the ordinary ether of the shops.

Dr. Battey stated that his remarks referred only to pure ether. The ordinary ether of the shops consists of ether, alcohol and water, and is more stable than Squibbs', which is very difficult to keep, from its great volatility. He uses a rubber cork after opening the hermetically sealed can. It fits tightly, and resists the action of the vapor of ether. The third objection urged against ether is its offensiveness to the patient.

Dr. Kendrick agrees with Dr. Baird. Has administered ether to feeble ladies, in whom it required thirty or forty minutes, or even an hour, and, in one case that he recalls, an hour and a quarter, to secure the full effect; on the other hand, has seen stout men come under its influence in ten minutes or less. Thinks it more active when the stomach is empty. Has observed that patients frequently resist until free vomiting occurs, when they rapidly succumb to its influence.

Dr. Armstrong concurs in the opinion that patients differ as to their susceptibility to anæsthetics; and also that ordinary ether is of very variable quality; agrees with Dr. Battey as to the proper method of administration. He entirely abandoned the use of chloroform years ago, except in convulsions. Never saw a death from ether, and has witnessed only one case in which it acted unpleasantly; a boy, much reduced by hemorhage from a wounded artery. As few whiffs of the ether suspended the circulation and respiration. He was resuscitated, and again he was overpowered in a few seconds. He was restored the second time, and the operation completed without the anæsthetic. Has so much confidence in the safety of ether that he scarcely considers it necessary to watch the pulse.

Dr. Kendrick thinks ether is not entirely free from danger. Saw a death from it, several years ago, in the Pennsylvania Hospital. The person was much worn by disease, and death occurred before the operation was commenced.

Dr. Battey: Does not think ether absolutely safe, but relatively so. Chloroform is very hazardous; ether slightly so. It, as a substitute for chloroform, reduces deaths by anæsthesia to the minimum. In answer to an inquiry, he said his idea of its action is that it produces an unnatural carbonization of the blood. The same result may be attained by carbonic acid gas, nitrous oxide gas, chloroform, nitrite of amyl, and other similar substances.

Dr. Bozeman has used chloroform a good deal, and seen it used extensively. Regards it a very dangerous remedy. Has seen one life sacrificed to it, and on several occasions has seen death produced, but the patients have been resuscitated. Ether is safer. Has never observed any unpleasant effects from the use of a mixture. Considers a judicious combination of a pure article, two or three parts of ether to one of chloroform, a great boon. In a large proportion of labors can use this mixture without danger and with decided advantage. Its use conserves the vital forces, but does not suspend the labor. In prolonged and painful surgical operations, thinks ether is not as efficient an anæsthetic as chloroform.

Dr. J. M. Johnson has heard a great deal said about the dangers of chloroform, but thinks, with Sir James Simpson, that a man who will not use it in obstetrical cases is almost a heathen. It may be given in a manner free from danger and productive of great good. When the os is rigid and the pains violent, fifteen drops on a handkerchief, held over the face of the patient, will afford quiet and relief, or when the perineum is unyielding and danger of rupture imminent, it should be used in the same way, with hot water in the bowel and warm application to the parts until relaxation is obtained. Often wrenching pains are felt in the back, without great uterine pains. Fifteen drops of chloroform, repeated as required, will relieve without opium, which he thinks should not be given. There are no pains greater than some of those that attend some labors, and can see no good reason why relief in this way should be withheld. Thinks the only danger is from the use of an excessive quantity.

Dr. Taliaferro is impressed with the danger of going to an extreme in abandoning the use of chloroform through fear of its effects. Uses it in labor when he deems it advisable. The intensity of the pains is moderated. Never knew of a death from chloroform in obstetrical practice, and, so far as his knowledge extended, none had been reported. In surgery prefers ether; considers it safer. Chloroform is preferable in obstetrics, as it acts quicker, and to secure its effects it is not necessary to saturate the patient with it.

Dr. Rauschenberg said within the last four weeks he had met with five cases of diphtheria, all occurring in crowded houses. Found, upon examination, the privies and water closets in bad condition and located very near the apartments of his patients. Thinks this fact goes to prove that there is danger from emenations from these sources. His treatment is quinine in large doses ten to twenty-four grains daily, according to the age of the individual; chromic acid, ten to fifteen grains to the ounce of water, topically, and carbolic acid internally. Thinks he is more successful in his treatment of this disease now than formerly.

---

J. B. BAIRD, M.D., REPORTER.

---

ATLANTA, June 1st, 1875.

Dr. J. M. Johnson, President, presiding.

Dr. Logan remarked, *apropos* of the discussion at the last meeting, that Dr. J. Marion Sims accounts for the freedom from disaster from the administration of chloroform during labor on the ground that the brain is full of blood, the effort during parturition causing cerebral congestion, and thus the tendency to syncope from the chloroform is counteracted.

Dr. Armstrong suggested that the small quantity employed was another explanation of the immunity from fatal results.

Dr. Woodhull said the objection to the last suggestion was the fact that when death occurred it often took place after a few whiffs only, and was not due to the quantity inhaled. He cited a case in point in which no organic lesions of the heart were found after death.

Dr. Armstrong thiuks that in such cases organic disease existed.

Dr. Taliaferro was under the impression that most physicians who use chloroform bring the patient fully under its influence; so much so that consciousness is abolished.

Dr. Logan concurred in this opinion. Thinks, from his experience and observation that no full effect is obtained unless it is carried to anæsthesia. This is claimed by the friends of the remedy.

Dr. J. M. Boring: Regards it necessary, especially in instrumental labors, that the patient should be as fully under its influence as in any surgical operation.

Dr. Rauschenberg has made very moderate use of chloroform in labor. He is opposed to its use, but has been persuaded to employ it in two cases. Unconsciousness was not produced, yet the patients were rendered comfortable. Does not see, when used to profound anæsthesia, how it can be continued through a protracted labor.

Dr. Armstrong was not aware that it was the custom of a majority of physicians to put the patient fully under its influence. It is not his habit to use it at all, unless some special indication arises. If his patient insists, gives a little, simply enough to lessen the intensity of the pain.

Dr. J. M. Johnson added a word in the line of his remarks at the last meeting. He invariably gives chloroform in labor, always in small quantity and always with the same result. Has never seen it used to total anæsthesia. If any gentleman has, should like to know what influence it exerted on the progress of labor. Even a small quantity sometimes arrests the pains. He knows that labor may go on during unconsciousness, as in convulsions, but knows no one bold enough to cause total anæsthesia and maintain it. Even when he administers it in doses of fifteen drops he places his finger on the pulse, and watches the respiration.

Dr. Logan, in order that he might not be misunderstood, desired to announce himself an opponent to the use of chloroform in labor. His experience is not very limited, and it is his observation that in the large majority of cases nothing is gained by its use. Unless sensibility is destroyed an excitement, physical, mental, moral and often immoral, is produced, that is very objectionable. Ordinarily confines its use to cases of difficulty. Thinks by it patients are rendered more liable to post partum hemorrhage. If the full effect is obtained nervous prostration is sure to follow.

Dr. Boring explained that he did not use it at all, except from necessity, and in his experience this necessity did not arise once in twenty cases.

Dr. Taliaferro said, in reply to the President, he would state that until the last few years he was in the habit of using it freely, to complete anæsthesia, in almost every case. Never saw a case in which the uterine contractions were retarded, or the action of the abdominal muscles suspended. Does not give it now to the same extent he formerly did; not because he is not an advocate of its use; he is; but on account of the big scare the profession is in about chloroform; fearing some accident may occur. If his patient requests chloroform she gets it. Has never seen the least unpleasant effect from it, either on the mother or the child. Should now use it to anæsthesia if necessary.

Dr. J. M. Johnson said Dr. Logan was the second gentleman he had ever heard speak of the danger of post partum hemorrhage following the use of chloroform. He cannot recall a single instance in which it has occurred; and if he should be called upon to express an opinion he would say directly the opposite.

Dr. Logan thought this fact was due to his manner of using it.

Dr. Johnson, by its use in very small quantities, encourages his patient, and affords her relief from pains that do not assist labor.

Dr. Connally has a few times used it to anæsthesia. It is his opinion that unpleasant effect was exerted upon the child.

Dr. Woodhull has had no experience himself, but in a recent conversation with a gentleman of extensive experience, he expressed the opinion that post partum hemorrhage was liable to follow its use. When he uses chloroform he always prepares himself for the emergency.

Dr. Armstrong reported the following case: Boy aged about fourteen years. When first seen had been sick for two weeks with continued fever and diarrhœa. Pulse 100. Tenderness in the ilio cæcal region. Regarded it a case of typhoid fever. No complications. Prognosis favorable. On 25th, three days after his first visit, was seized with obstinate vomiting. The matter ejected was green and bitter. On the morning of the 26th a tumor in the abdomen developed in the course of a few hours. It was about the width of four fingers, and extended from the symphysis pubis obliquely upwards and outwards almost to the costal cartilages. No unusual tenderness; symptoms unchanged. An exploring needle was introduced into the tumor on the 27th, but without result. Was unable to arrive at any satisfactory diagnosis. Thought it might have been produced by discharge of a hepatic abscess, or, what was more likely, an acute abscess of the abdominal walls, or possibly peritonitis. On 28th, with the assistance of Dr. J. M. Johnson, the tumor was tapped, and about twenty-four ounces, of watery, slightly colored fluid drawn off by means of an aspirator. Constant vomiting continued. He died on the 30th. No post-mortem could be obtained. The fluid was subjected to microscopical examination by Dr. Baird, who, by request, stated that it contained blood corpuscles and a few tessellated epithelial scales. No albumen. He regarded it a case of peritonitis, due to perforation; a spontaneous development. Pain, as is well known, in some cases of peritonitis is wanting; and even marked tenderness in rare cases is absent.

Dr. J. M. Johnson mentioned that purpuric spots were observed on the abdomen four days before death. Concurs in the opinion that it was peritonitis, and thought, from the absence of pain, that it was sub-acute.

Dr. Battey presented an ovarian tumor removed the day before from a young lady aged sixteen years; the same person from whom one of the samples of cystic fluid, presented a few weeks ago, was obtained. His diagnosis was multilocular cystic tumor of the right ovary, free from adhesions. This diagnosis was verified at the operation, except that it was the left instead of the right ovary. Found the contents of the principal cyst identical with that exhibited to the Academy—a limpid, slightly albuminous fluid. In the other large cyst the fluid was syrupy in consistency, and of a straw color. In another it was stringy like honey, and in others gelatinous. After removing the tumor, found the right ovary very much enlarged, and in removing it a cyst containing two drachms of fluid was opened, and upon examination its surface was found to be covered with little cysts containing galitiniform matter. The left fallopian tube was greatly enlarged and hypertrophied. The tumor contained fully fifty cysts, and its estimated weight was twenty-two to twenty-four pounds. The amount of fluid withdrawn filled an ordinary water bucket. Has progressed fairly since the operation, though the reaction is excessive. Following morning pulse 120; temperature 103°. Evening pulse 140. Small doses of morphine.

Dr. J. M. Johnson said he witnessed this operation for the first time, and with pleasure. It is a terrible one, and he must say it was splendidly performed. It is the crowning achievement in surgery. Adjourned.

---

ROBERT BATTEY, M.D., REPORTER.

---

ATLANTA, 8th June, 1875.

The President, Dr. John M. Johnson, presiding.

Dr. Logan stated that Simpson was in the habit of inducing sleep by chloroform in the first stage of labor, and in the second stage pushed it habitually to entire insensibility.

Dr. John M. Johnson was of the opinion that the accepted rule of the profession at present is to give only small quantities of chloroform in labors.

Dr. Taliaferro alluded to the method of "breaking the suction," as illustrated by Prof. H. F. Campbell at the Georgia Medical Association, by means of his "pneumatic self-repositor." He suggested the use of two fingers passed into the vagina, and the perineum to be drawn backwards, when the air rushes in at once. He also suggested a similar proceeding in the rectum, distending this tube also with air to more effectually reposit the fundus uteri.

Dr. Logan suggested that the appliance of Dr. Campbell might be considered more delicate and elegant than the use of the fingers. He also thought that the tube might be more efficient in its action.

Dr. Taliaferro thought there could be no additional force exerted by the use of Dr. Campbell's tube.

Dr. Battey remarked that Dr. Campbell had called attention to the use of the fingers, as suggested by Dr. Taliaferro, but had devised the tube as a more delicate and elegant means.

Dr. John M. Johnson had used the method of Dr. Campbell with satisfaction. He thought the tube of Dr. C. threw around the proceeding a higher dignity, so to speak.

Dr. Westmoreland asked how long the womb would remain in position after being thus reposited.

Dr. Johnson replied that he directs the patient to remain for a time on the knees, and then quietly and gently recline upon the side, the hips being supported a little by a small pillow, and so remain for the night. He finds this method in most cases, greatly superior to the use of pessaries. Does not deny the utility of pessaries, but greatly prefers the method described to pessaries.

Dr. Westmoreland thought that we could not keep a patient either upon the knees or upon one side for three or four months.

As soon as the patient moves or gets up, the air escapes, and the uterus is again displaced. How is it that we may expect cures from this course of treatment?

Dr. Taliaferro understands that the temporary replacement of the uterus enables the organ to become gradually accustomed to its normal position, until it gains strength and is better fitted for the use of a pessary.

Dr. Battey explained the views of Dr. Campbell. In the first place, many of these uterine displacements are caused by, and attended with, uterine disease, usually of inflammatory character. And secondly, there is relaxation and debility of uterine supports. The irritable and inflamed uterus often will not bear the support of a pessary, when it will bear the cushioning of an inflated vagina with the greatest ease and comfort. The uterine supports are relieved from tension for hours every night, and thus enabled to regain in part their lost tonicity. Dr. Campbell designs by the method to supplement pessaries and to prepare the patient for the use of the pessary, rather than to discard that appliance entirely. The patient may retain the uterus *in situ* for an entire night, and during the day too, if the case compels the patient to be bed-ridden. If in change of position in bed the air escapes, it is only required to reposit the uterus again and recline upon the opposite side.

Dr. W. F. Westmoreland reported case of a lady who at thirteen years old, at a boarding school, had trouble in the establishment of the menses. She was quite hysterical. She was taken from school and soon recovered. At the age of twenty-one she again came under his care as a married woman of two years. She was now menstruating only once in four to eight or nine months. Examination revealed an infantile uterus of not more than half size. There was much nervous disturbance and impairment of general health. He resorted to uterine tents, and the uterus rapidly developed, with return of the menses every two or three months, and subsidence of all the nervous symptoms. Latterly the menses recur only once in six or eight months. She again has the nervous symptoms. She is in good flesh, in fact is fat. The uterus appears to be now entirely normal. He asks, whence now the amenorrhœa? Is it uterine or ovarian? What the remedy? She has not menstruated now for five or six months.

Dr. Battey suggested that the cause of failure of the menses here is likely due to some defect in the ovaries, of which we know little or nothing, but which he hopes he has put the profession now in a position to investigate. When we say the case is one of atony of the uterus, we merely beg the question, and really state nothing which throws light upon its pathology. He thinks the more hopeful line of treatment will be by local galvanic action of Simpson's stem pessary. It has been doubted whether any galvanic action results from the use of this instrument. There can be no rational doubt. The shock elicited from a disk of zinc and one of silver, in the mouth, goes to prove it; also the rapid chemical action of the uterine secretions upon the zinc element establish the fact most clearly. The galvanic stem, doubtless, influences the ovaries as well as the uterus.

Dr. Taliaferro thought the suggestion of the galvanic stem the proper treatment of the case. The nervous connection between the ovaries and uterus is very intimate, and the galvanic action of the stem upon the ovaries is undoubted.

Dr. Johnson thought that the general health ought to be looked to, and marriage instituted and relied upon. Does not approve of the use of tents and stem pessaries. He does not believe that the ovaries have any connection with menstruation. Thinks the ovaries have no more connection with the function of the uterus than with the functions of the rectum or bladder. If marriage and nature's pessary does not cure the case, no other pessary will.

Dr. Battey reported the fatal issue of the case from which he removed the large multilocular cyst of the left ovary, and also the similarly degenerated right ovary, on Monday of last week, and remarked:

It is often highly instructive to retrospect the history of cases which have resulted fatally in our hands, and mark the lessons which they teach. In this case we had presented an assemblage of conditions seemingly most favorable for an operation. The subject was young and of vigorous constitution—a well developed mountain girl of barely sixteen—in good state of general health, with a tumor developed to the third stage, free of adhesions, with a long and thin pedicle, and no previous symptoms of inflammatory action in the tumor or peritoneum. The operation passed off as smoothly as the movements of a watch. The patient rallied with the minimum of shock, slept quietly under very small doses (one-sixth grain) of morphia, repeated at intervals of five to eight hours. The large cyst had been drawn out, by successive tappings, through an incision of not more than four and a half inches in the perieties of the abdomen; none of its contents were allowed to enter the cavity. The degenerate right ovary had been brought to the surface, its base transfixed and securely tied, and the ovary cut away, when the ligatures were cut short and the stump dropped free into the pelvis. A clean sponge was twice passed down into the bottom of the pelvis and returned with but a slight stain upon it. The main stump was clamped and lodged in the lower angle of the incision, exerting no traction whatever upon the uterus, which remained in its normal position. So far all seemed as lovely as the bright May morning which greeted the operation.

At 9 P.M. the pulse was 100, the patient resting quietly and sleeping most of the time. On the morning of the second day the reaction was excessive; the pulse 120, the temperature 103° In the evening the pulse had mounted to 140—the temperature remaining 103°. Third day morning there was little billious vomiting, the pulse fell to 120, the temperature to 101°. In the afternoon the pulse mounted to 148, and temperature to 103°. It was deemed advisable to control the excessive action of the heart, and Norwood's Veratrum was given in small doses (11 drops in all) through the night. By daylight of the fourth day the pulse was reduced to 88, and the extremities were very cold and clammy. At 8 A.M. the pulse stood 108, temperature 102°. At noon, extremities still cool, pulse 120, temperature 101°; at 7 P.M., still cold and clammy; pulse 120, temperature 101°; at 9 P.M. still cold, pulse 120, small and fluctuating. During the day she asserted that she was well; desired to get up; very restless, complaing of heat; mind excited as if in slight intoxication. At midnight the pulse was still 120, but more full and steady, and the extremities had warmed up. This improvement, however, was but temporary; soon she declined again, and rapidly fell into collapse towards morning, and died at 10 A.M. of the fifth day. So decided were the symptoms of acute septicæmia it was deemed advisable, two hours before death, to pass a double catheter down by the pedicle into the pelvis, and inject warm water into the cavity, which returned without evidence of septic fluid.

Autopsy was held two hours after death by Dr. J. T. Johnson, in presence of Dr. W. F. Westmoreland. Dr. Westmoreland reports:

Autopsy two hours after death. Upon opening the abdomen the first glance showed the existence of general peritonitis. The omentum was glued to the bowels by the lymph that was thrown out, which was partly organized. The pedicle had nearly healed; the stump doing well. In both iliac regions, and in Douglas' fossa, bloody serum, containing flakes of lymph, was found; about six ounces in all. These effusions were circumscribed by adhesions and lymph. The inflammation was more intense in the pelvic than in the abdominal peritoneum. The fluid was very acrid, being extremely irritating to the hands, and stinging the fingers during the examination.

Dr. Battey remarked: The cause of death here, viewed in the light of the history and the post-mortem revelations, is clearly a blood poisoning by the absorption of the extremely acrid and highly irritating fiuid in the pelvic cavity. The death is not by peritonitis, but by acute septicæmia, by reason of the absorption of this highly septic material and its poisonous action upon the vital centres.

He has observed, after operations in the peritoneal cavity, and especially in normal ovariotomy, that there is promptly poured out a serous exudation from the irritated peritoneum, which, in the latter case, when the incission passes up through the vaginal cul-de-sac, escapes into the vagina and externally. There is reason to believe that such exudation, not the result of peritonitis, but simply of irritation of the serous membrane, occurs too in the major operation, and, in the case before us, also some little bloody fluid, perhaps, oozing from the second stump, which had been dropped back into the pelvis. These fluids, which in the vaginal section would escape at once through the incission, are of course pent up within the cavity, and may be disposed of in three ways, or, rather, lead to either of three terminations, i.e.: First, being small in amount, the fluid doubtless is subject to absorption and removal by natural laws. Secondly, by reason of the failure of absorption, by its tardiness, or by some peculiar character, as yet unknown, of the fluid itself, it undergoes decomposition, and septic material results. In such case the absorption of the septic product would likely give rise to symptoms of acute septicæmia, such as elevation of the pulse to 120, 140 or more, the pulse being smaller, softer and weaker than that of acute peritonitis; also clammy sweats, high temperature in the axilla, coldness of the extremities, etc, and *especially*

a certain restlessness and excited state of the brain, with more or less of intoxication, and the absence of any meteorism of the abdomen, and of any considerable pain or abdominal tenderness. In such circumstances, the amount of septic material being small, the process of absorption may go on to the entire elimination of of the effused fluid, and recovery result. Or, the patint dying subsequently of some intercurrent disease, the peritoneal cavity may be found free of fluid and healthy. And thirdly, the septic material being too great to be disposed of in this way, the adjacent peritoneum inflames, and the patient succumbs with symptoms of decided blood poisoning, or, in other words, of acute septicæmia.

When in these fatal cases the autopsy shows the co-existence of abundant acrid septic material, with peritoneal adhesions and other inflammatory changes, a distinction is to be drawn from other cases wherein the previous history has shown the evident symptoms of grave peritonitis, with hard, wiry pulse, ballooning, and great tenderness of the abdomen, etc., and the autopsy reveals no fluid exudation, or one of a non-septic character. In the former the essential disease is acute septicæmia, and the death is by blood poisoning of the vital centres. In the latter the essential element is acute peritonitis, and the death is attributable to that cause. In the two the rational signs are strikingly different, and the manner of the death is also different.

It is the more important to draw this distinction because in the one case the prognosis is exceedingly bad, whilst in the other we may indulge more rational hope that the patient will "pull through." It is vitally important for rational treatment, since we may indulge hope of successful issue of acute peritonitis under the profound tranquilization of opium; whilst in acute septicæmia we are only adding the opium poisoning to cloud the deadly septic poisoning which is hurrying the patient to destruction. It is true that a limited amount of septic material may be eliminated by nature's powers, but in the great mass of instances the quantity far transcends these narrow limits, and a riddance of the poison by art offers the only rational ground for hope.

Taking this view of the subject it would seem to be good practice, when the pulse mounts up rapidly and to the excessive degree it did in the case under consideration, with the distinctive signs of septic poisoning, to introduce a double catheter through the abdominal wound *at once* and into the more dependent pelvic cavity, and cleanse it thoroughly with simple water at blood heat, which may be made to flow in and out again until the thorough cleansing is accomplished; and even should there be mistake in the discriminating diagnosis it would seem that no harm could result, but good rather, in bathing an inflamed peritoneum with simple tepid water.

It has been proposed by Dr. J. Marion Sims to make an opening at each operation through the botton of the *cul-de-sac*, and introduce a drainage tube through the vagina. Dr. Thomas also suggests and practices the lodgment of a glass tube in the lower angle of the incision, which tube extends to the bottom of the *cul-de-sac*. In both suggestions the operation is complicated with a source of irritation in the foreign body used, and in the first suggestion an additional incision also into the peritoneum. No difficulty need be experienced in the introduction of the double catheter through the abdominal incision and deep down into the pelvis whenever occasion may require, by rupturing recent adhesions if need be for this purpose.

In the present case, it is to be remarked that the septic symptoms set in upon the second day, a very early period for such a condition; a period when we would expect acute peritonitis, and not septicæmia. The authorities all agree in the opinion that the source of danger in the first four days is peritonitis; here, however, the early symptoms pointed clearly to septic poisoning, and not peritonitis, and it may well be asked if prompt steps had been taken on the afternoon of the second day to determine the question definitely, and to thoroughly wash out the cavity, would not the issue of the case have been widely different? The attempt to wash out the cavity on the fifth day morning was fruitless, because the septic material had become encapsuled by broad bands of lymph, cutting it off from the general cavity.

Adjourned.

---

To Prevent Marks of Small-Pox.—Touch the postules with glycerin, and then sprinkle with a powder of red oxyd of mercury one part, sublimed sulphur four parts. An Italian physician says this will cause the postules to dry and desquamate in a few days, without leaving the slightest mark.—*Pacific Medical nd Surgical Journal.*

